# Influence of age on clinical presentation, diagnosis delay and outcome in pre-school children with acute appendicitis

**DOI:** 10.1186/s12887-020-02053-5

**Published:** 2020-04-06

**Authors:** Yasmine Lounis, Julie Hugo, Martine Demarche, Marie-Christine Seghaye

**Affiliations:** 1 Department of Pediatrics, University Hospital Liège, Liège, Belgium; 2 Department of Emergency Medicine, University Hospital Liège, Liège, Belgium; 3 Department of Pediatric Surgery, Regional Hospital Citadelle, Liège, Belgium

**Keywords:** Acute appendicitis - children, Clinical presentation- diagnosis, Complications, Perforation

## Abstract

**Background:**

Unusual clinical presentation of acute appendicitis in preschool children leads to misdiagnosis and complications.

We aimed to analyze the influence of age on clinical presentation, laboratory findings and complications in preschool children with acute appendicitis.

**Methods:**

From January 2012 until December 2017, 29 children younger than 6 years of age (median 50 months) with acute appendicitis were enrolled in this retrospective study. Patients were grouped according to their age: group 1: < 48 months (*n* = 13); group 2: > 48 months (*n* = 16), their clinical data, laboratory results and complications were compared.

**Results:**

In group 1, duration of nausea and vomiting was longer, alteration of general state was more frequent and pain in the right fossa iliaca less frequent than in group 2 (*p* = 0.026, *p* = 0.000 and *p* = 0.029, respectively). Heart rate was higher in group 1 than in group 2 (*p* = 0.012). Leucocyte and polynuclear neutrophil counts were lower in group 1 than in group 2 (*p* = 0.028 and = 0.004, respectively) but C-reactive protein levels were not different between groups. In the whole cohort however, C-reactive protein at admission value correlated negatively with age (*p* = 0.025).

Abdominal ultrasound allowed diagnosis in 19/29 patients (65.5%), without any difference between groups. Appendicular perforation was more frequent in group 1 than in group 2 (*p* = 0.003). Perforation was also related to longer hospital stay (*p* = 0.018). Peritonitis occurred in 21/29 (72%), post-operative ileus in 5/29 (17%) and sepsis in 4/29 (14%) patients without any difference between groups. In the whole cohort, hospital stay correlated negatively with age (*p* = 0.000). There was no mortality.

**Conclusions:**

Among preschool children, those younger than 48 months present with longer duration of pre-admission symptoms indicating longer infection course than in older children. Altered general state and higher degree of tachycardia in the younger reflect higher systemic repercussions of the illness. Less specific abdominal pain and dissociation of the inflammatory markers with lower leucocyte- and neutrophil counts and higher C-reactive protein levels in the younger may contribute to further diagnosis delay and higher rate of perforation in these patients.

## Background

Acute appendicitis is rare condition in children under 6 years of age and is often diagnosed with delay in this age group [1]. Indeed, an initial diagnostic error rate ranging from 28 to 57% is reported in children 12 years old or younger and can reach 100% in those 2 years of age or younger [2]. A recent study showed a significant increase of perforation in relation with age as follows: 100% < 1 year; 100% 1–2 years; 83,3% 2–3 years; 71,4% 3–4 years; 78,6% 4–5 years and 47,3% 5 years [3].

The diagnostic delay is partly due to unclear anamnesis and atypical clinical presentations found in two-thirds of these young patients [4]. The most frequent diagnosis in young children who are primary examined in the context of abdominal pain with vomiting and diarrhea and in whom acute appendicitis is finally diagnosed is acute gastro-enteritis [5].

This misdiagnosis is due to the fact that the classical clinical symptoms and laboratory findings that are the rule in older children and adolescents are missing in the younger [6].

The banality of acute gastro-enteritis and the reinsurance of caregivers delay appropriate surgical treatment, explaining higher rate of complications in younger children [7]. Besides diagnosis and treatment delay, appendicitis occurs on a particular terrain in children characterized by the fragility of the appendicular wall and by the relative immaturity of the large omentum. This makes the condition more critical and more prone to complications in a younger patient [8].

In the pediatric population, complicated intra-abdominal infections are, in most of the cases, caused by perforation of the appendix and may be one of the most important causes of morbidity [9, 10]. Thus, in children under 6 years of age two-third of appendicitis are complicated [11] with a perforation rate ranging from 57 to 100% in children younger than 4–5 years and 1 year of age, respectively [12].

The aim of this retrospective study was to analyze the incidence of primary symptoms, clinical- and laboratory parameters and complications in a cohort of preschool children younger than 6 years of age in whom acute appendicitis was diagnosed. The focus of the study was set on the influence of age on the outcome variables.

## Methods

The Ethics Committee of the University Hospital of Liège approved this retrospective study.

Inclusion criteria: all children of both genders younger than 6 years of age operated for acute appendicitis between January 2012 until December 2017 in our department.

Exclusion criteria: all children who did not fit the inclusion criteria or in whom the patient file was incomplete.

Between January 2012 and December 2017, 369 children younger than 16 years of age were admitted in our emergency department for acute appendicitis and underwent appendectomy. Thirty-four (8,9%) were preschool children, younger than 6 years of age, 5 of them were excluded because of incomplete patient records. The remaining 29 were eligible for the study. The number of cases pro year was as follows: 2012: *n* = 4; 2013: *n* = 2; 2014: *n* = 5; 2015: *n* = 8; 2016: *n* = 6; 2017: *n* = 4.

Based on patient age distribution, patients younger than 48 months (*n* = 13) were assigned to group 1 and those older than 48 months (*n* = 16) to group 2.

Pediatricians and nurses of our institution had access to an electronical patient record that precisely documents patient history including suspected diagnosis and symptoms (type and duration of abdominal pain and its localization, nausea and vomiting, diarrhea, anorexia), demographic data (gender, age, weight, body mass index (BMI)), rectal temperature, quality of the general state, hemodynamic data (heart rate, blood pressure, capillary refill time) and a complete examination of all organ systems.

**Fig. 1:**
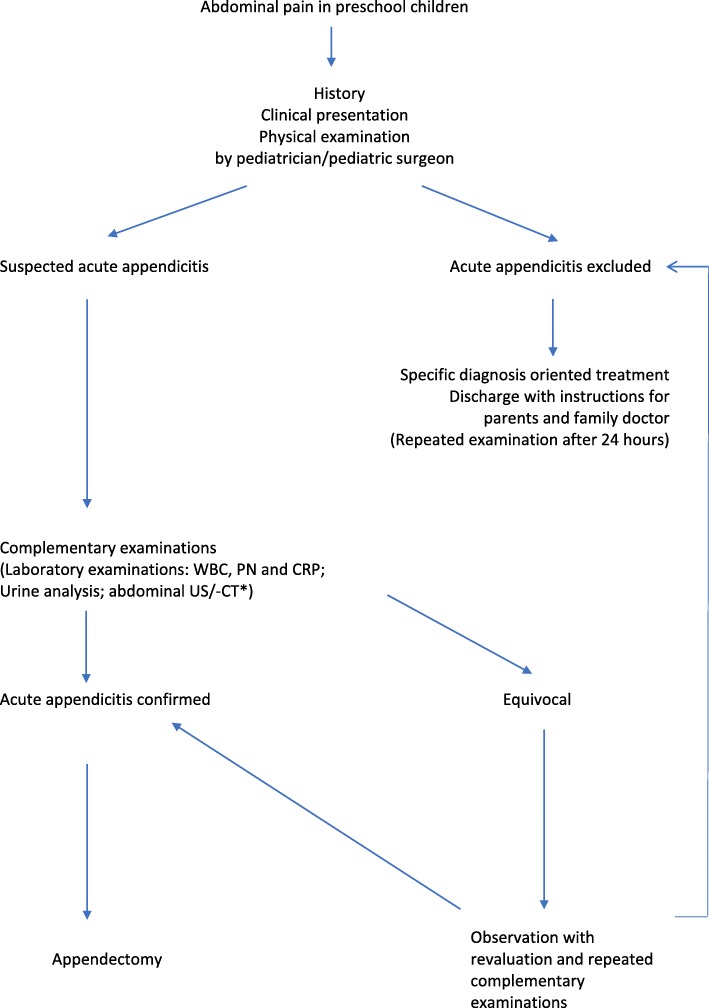
Algorithm of the diagnostic procedure and treatment in preschool children with abdominal pain. WBC: white blood cell; PN: neutrophil; CRP: C-reactive protein; US: ultrasound; CT: computed tomography. *: only in equivocal cases

All patients were admitted for abdominal pain and were managed according to an algorithm helping to diagnose or exclude appendicitis (Fig. 1).

Pre-operative laboratory examination including determination of white blood cell (WBC)- and polynuclear neutrophil (PN) count and C-reactive protein (CRP) blood levels was performed in all patients. All patients underwent pre-operative imaging by abdominal ultrasound and abdominal computed tomography (CT)-scanner if necessary. Direct signs of appendicitis on ultrasound were thickening, hyperemia, and incompressibility of the appendix, layer dedifferentiation and presence of an appendicolithis. Indirect signs were peri-appendicular fat infiltration, mesenteric adenomegaly and reactive peritoneal effusion.

The Pediatric Appendicitis Score (PAS) [13] and the score of Alvarado [14] were assessed in each case retrospectively, according to a previous study [15].

Urgent or scheduled appendectomy was performed either by laparoscopy or by laparotomy, depending on surgeon’s preference.

Complications such as appendix perforation, peritonitis (inflammation of the peritoneum with or without purulent peritoneal liquid), or abscess formation were diagnosed by ultrasound, at surgery and confirmed by histological analysis.Children with perforated appendix, peritonitis or persisting post-operative fever received intravenous antibiotics (amoxicillin/clavulanic acid (100 mg/kg/day) with or without metronidazole (30 mg/kg/day)) for up to 5 days. This was followed by oral antibiotherapy (amoxicillin/clavulanic acid (50 mg/kg/day) or cefuroxime (50 mg/kg/) for 5 more days. In case of sepsis associated with perforation or peritonitis, the switch to broad spectrum intravenous antibiotics was undertaken for 10–15 days (piperacillin 100 mg/kg/day/tazobactam 12.5 mg/kg/day, or ceftazidime 50 mg/kg/day). Oral relay was undertaken as described above or with ciprofloxacin (20 mg/kg/day) for up to 10 days. According to the clinical response to treatment, antibiotherapy was implemented by amikacin (15 mg/kg/day), ampicillin (50 mg/kg/day), glazidim (50 mg/kg), or vancomycin (60 mg/kg/day, 6H).

Children were discharged as soon as they were in good general state, afebrile, painless and with feeding autonomy.

Primary outcome variables were duration of pre-admission symptoms that means the time period between presentation of the first symptoms of appendicitis and admission, clinical presentation and laboratory findings. Secondary outcome variables were incidence of operative complications and duration of hospital stay and were analyzed by comparison of both patient groups and with respect to the presence of appendicular perforation.

### Statistical analysis

Data were analyzed by the Statistical Package for The Social Sciences SPSS 22,0, IBM corporation, Armonk, USA.

Results are shown by the median and interquartile range (IQR), according to the non-normal data distribution.Inter-group comparison of the median values was performed by the non-parametrical Mann -Whithney U test, distribution of categorical variables by the chi-square test and correlation analysis by calculating the Spearman rank correlation coefficient.

*P*-values < 0.05 were considered significant, *p*-values < 0.1 indicated a tendency toward significance.

## Results

Demographic-, clinical patient data and laboratory results are summarized in Table 1.

**Table 1:** Demographic, Clinical and laboratory data in all patients, in group 1 and in group 2

	All (*N* = 29)	Group 1 (*N* = 13)	Group 2 (*N* = 16)	P
**Demographic data**				
Age (months)	50 (25.5)	35 (10)	59 (16.25)	0.000
Gender				0.43
Male (n)	14	7	7	
Female (n)	15	6	9	
Weight (kg)	16 (5.25)	14.5 (2.55)	18.8 (6.37)	0.000
BMI (kg/m^2^)	15.4 (4.1)	14.5 (2.85)	16.1 (4.55)	0.23
**Pre-admission symptoms**				
Overall duration of symptoms (h)	48 (72)	72 (72)	24 (48)	0.056
Duration of abdominal pain (h)	36 (84)	62 (84)	19 (46.5)	0.061
Duration of nausea-vomiting (h)	31 (66)	72 (90)	24 (12.5)	0.026
Duration of fever (h)	48 (54)	67 (69)	30 (51)	0.19
**Clinical data**				
Temperature at admission (°C)	37.5 (1.8)	37.8 (1.4)	37 (1.1)	0.062
Heart rate at admission (bpm)	130 (42.5)	153 (32)	120 (36.7)	0.012
Maximal temperature (°C)	38.8 (1)	38.9 (1.1)	38.6 (1.08)	0.33
**Laboratory data at admission**				
Leukocyte count (×10^9^/L)	17.2 (9.08)	12.2 (8.94)	17.9 (5.94)	0.028
Neutrophil count (×10^9^/L)	12.9 (7.27)	9.6 (5.72)	14.9 (2.97)	0.004
CRP (mg%)	121.9 (145.2)	134 (140)	67.3 (146)	0.13
**Scores**				
PAS score (/10)	5 (2.5)	5 (2)	5 (3)	0.91
Alvarado (/10)	5 (3.5)	5 (2.5)	5.5 (4.5)	0.45
Duration of hospital stay (d)	6 (4)	7 (5)	3 (3.75)	0.067

Data are shown by the median value and (interquartile range). Group 1: < 48 months; Group 2: > 48 months

Table 2 shows the incidence of outcome variables in both patient groups.

**Table 2:** Incidence of outcome variables in both patient groups

	All patients *N* = 29	Group 1 *n* = 13	Group 2 *N* = 16	P
Pain right fossa iliaca	11 (34.4%)	2 (15.4%)	9 (56.2%)	0.029
Alteration general state	16 (55.1%)	12 (92 .3%)	4 (25.0%)	0.000
Purulent peritoneal liquid	11 (34.4%)	8 (61.5%)	3 (18.7%)	0.023
Perforation	11 (34.4%)	9 (69.2%)	2 (12.5%)	0.003

Statistical analysis was performed by the χ^2^ test. Group 1: < 48 months; Group 2: > 48 months

In the whole cohort, age was 50 (25,5) months (group 1: 35 (10); group 2: 59 (16,25) months, respectively).

Median overall duration of symptoms before admission was 48 h (h) (72) in the whole group. It tended to be longer in group 1 (72 h (72)) than in group 2 (24 h (48)), *p* = 0.056).

Duration of nausea/vomiting before admission was significantly longer in group 1 than in group 2 (72 h (90) versus 24 h (12,5), respectively) (*p* = 0.026), whereas duration of abdominal pain tended to be longer in group 1 than in group 2 (62 h (84) versus 19 h (46,5), respectively, *p* = 0.61). Duration of fever was not different between groups.

Upon apparition of the first symptoms and before admission in the emergency department, 17 patients (59%) had had an ambulatory examination. Diagnosis of acute appendicitis was made in only 5 of them. In the remaining 12 patients, diagnosis was acute viral gastro-enteritis (*n* = 4), urinary tract infection (*n* = 3), constipation (*n* = 2), viral infection (*n* = 2) and bronchitis (*n* = 1). There was no difference between groups.

At admission, alteration of the general state was present in 16 children and was more frequent in group 1 than in group 2 (*p* = 0.000).

All patients complained about abdominal pain that was diffuse (*n* = 18) or located in the right fossa iliaca (*n* = 11). This later was less frequent in group 1 than in group 2 (*p* = 0.029).

The majority of the patients showed anorexia (*n* = 20), fever (*n* = 18), nausea and/or vomiting (*n* = 16). Eleven patients showed diarrhea, 11 constipation and 6 painful urination, without any difference between groups.

Temperature at admission tended to be higher in group 1 than in group 2 (37,8 °C (1,4) versus 37 °C (1,1), respectively, (*p* = 0.062), whereas heart rate was significantly higher in group 1 than in group 2 (153 bpm (32) versus 120 bpm (36,7), respectively, *p* = 0.012).

At admission 17 patients (59%) showed increased WBC -, 23 patients (79%) increased PN count and 25 patients (86%) increased CRP. Fifteen children (52%) had a combination of hyperleukocytosis and increased CRP. Only one patient (3%) has no increased inflammatory markers.

WBC- and PN count were significantly lower in group 1 than in group 2 (WBC: 12.2 × 10^9^/L (8,94) versus 17.9 × 10^9^/L (5,94), respectively, *p* = 0.028; PN: 9.6 × 10^9^/L (5,72) versus 14.9 × 10^9^/L (2,97), respectively, *p* = 0.004). CRP concentration was not different between groups.PAS score was positive in 12 patients (41%). Alvarado score was compatible with appendicitis in 9 children (31%), suggested probable or very likely appendicitis in 7 children (27) % and 1 child (3%), respectively.

PAS- and Alvarado scores were not different between groups. Only 19 out of all patients (65.5%) displayed either direct or an association of direct and indirect signs of appendicitis at this examination (group 1: *n* = 9; group 2: *n* = 10, *p* = 0.63). Abdominal CT-scan was performed for diagnosis confirmation in 6 patients in whom second ultrasound was not contributive (group 1: *n* = 1; group 2: *n* = 5, *p* = 0.18).

Surgery took place either as immediate emergency intervention or was scheduled at admission or not later than in the early next morning if diagnosis was achieved in late night in the majority of the cases (*n* = 25; 86%). In 4 cases (group 1: *n* = 1; group 2: *n* = 3), surgery was delayed until diagnosis confirmation or because of misdiagnosis and finally performed as emergency. Three out of these patients had appendicular abscess and perforation. Six patients had retro-caecal appendix (*n* = 3 each group).

Eighteen (62%) patients underwent laparoscopy and 11 (38%) laparotomy, 4 of them after open conversion (14%). Operation technique was not different between groups.

At operation, perforation was reported in 9 patients of group 1 and in 2 of group 2 (*p* = 0.003). Peritoneal liquid was purulent in 8 patients of group 1 and in 3 of group 2 (*p* = 0.023). Peritonitis was the most frequent intra-operative finding in the whole cohort (72%). In 5 patients post-operative ileus occurred. Treatment consisted of antalgic control, bowel rest, gastric liquid aspiration and intravenous infusion of a crystalloid solution for hydration until bowel transit recovered after a median delay of 2 days (IQR: 1.5 days). Alizapride chlorhydrate was given as anti-emetic medication if necessary.

Twenty five children received intravenous combination of amoxyciline/clavulanic acid that was associated with metronidazole in 17 and followed by an oral relay, according to our protocol.

Four children developed sepsis and required a broad spectrum antibiotherapy for a median duration of 16 days (IQR: 6.5 days): One patient received the association of intravenous amoxyciline/clavulanic acid and metronidazole followed by oral amoxyciline/clavulanic acid for a total of 10 days; One patient received intravenous amoxyciline/clavulanic acid and metronidazole followed by oral ciprofloxacin for a total of 17 days; one patient received intravenous amoxyciline/clavulanic acid and metronidazole for 5 days that was switched to intravenous piperacillin/tazobactam for 5 days, followed by oral ceftazidime for 5 more days (total 15 days). The last patient did not respond to the initial intravenous amoxyciline/clavulanic acid and metronidazole association that was enlarged with intravenous amikacin. The treatment was switched to glazidim, ampicillin, vancomycin and metronidazole 2 days later for a total of 18 days. Oral treatment consisted of amoxyciline/clavulanic acid and ciprofloxacin for 11 more days.

Two patients required a second surgery. There was no difference in post-operative complications between groups. Complications are summarized in Table 3.

**Table 3:** Complications of acute appendicitis in all patients and in both patient groups

	All patients (*N* = 29)	Group 1 (*N* = 13)	Group 2 (*N* = 16)	P
Peritonitis	21 (72%)	11 (85%)	10 (62.5%)	0.18
Appendicular abscess	12 (41%)	7 (54%)	5 (31%)	0.19
Perforation	11 (38%)	9 (69%)	2 (12.5%)	0.003
Post-operative ileus	5 (17%)	3 (23%)	2 (12.5%)	0.39
Sepsis	4 (14%)	1 (8%)	3 (18.7%)	0.38
Second surgery	2 (7%)	1 (8%)	1 (6%)	0.70

Statistical analysis was performed by the χ^2^-test. Group 1: < 48 months; Group 2: > 48 months

Length of hospital stay was 6 days (4) and tended to be longer in group 1 (7 (5)) than in group 2 (3 (3.75)) (*p* = 0.67). In the whole cohort, it correlated negatively with age (Spearman rank correlation coefficient − 0.668, *p* = 0.000) (Table 4). Patients with perforation had a longer hospital stay than the others (7 days (4) versus 3.5 (3,75), *p* = 0.018) (Table 5).

**Table 4:** Correlations between patient age and outcome variables in the whole cohort (*n* = 29)

	Spearman rank coefficient	P
Age versus		
* Overall duration of pre-admission symptoms	−0.495	0.007
*Duration abdominal pain before admission	−0.422	0.028
*Duration nausea-vomiting before admission	−0.531	0.034
*Temperature at admission	−0.527	0.003
*Heart rate at admission	− 0.627	0.000
*White blood cell count at admission	0,315	0,096
*Neutrophil count at admission	0.442	0.016
*CRP-value at admission	−0.416	0.025
*Maximal CRP-value	−0.345	0.067
*Duration of hospital stay	−0.668	0.000

Statistical analysis was performed by the Spearman rank test

**Table 5:** Demographic, Clinical and laboratory data in all patients, in patients with and without perforation

	All (*N* = 29)	Perforated (*N* = 11)	Non-Perforated (*N* = 18)	P
**Demographic data**				
Age (months)	50 (25.5)	35 (16)	58 (22,2)	0.002
Gender				0.33
Male (n)	14	6	8	
Female (n)	15	5	10	
Weight (kg)	16 (5.25)	15 (2.51)	18.8 (8,5)	0.002
BMI (kg/m^2^)	15.4 (4.1)	14.5 (2.6)	16.1 (4.55)	0.025
**Pre-admission symptoms**				
Overall duration of symptoms (h)	48 (72)	72 (52)	32 (53)	0.14
Duration of abdominal pain (h)	36 (84)	55 (96)	24 (51,5)	0.069
Duration of nausea-vomiting (h)	31 (66)	72 (90)	24 (32,5)	0.33
Duration of fever (h)	48 (54)	67 (96)	36 (51)	0.44
**Clinical data**				
Temperature at admission (°C)	37.5 (1.8)	37.8 (1.2)	36,8 (3,4)	0.31
Heart rate at admission (bpm)	130 (42)	153 (25)	118,5 (41)	0.008
Maximal temperature (°C)	38.8 (1)	38.6 (1.0)	118,5 (41)	0.008
**Laboratory data at admission**				
Leukocyte count (×109/L)	17.2 (9.08)	17.24 (11.93)	17.1 (8.74)	0.52
Neutrophil count (×109/L)	12.9 (7.27)	12.14 (7.34)	14.0 (7.65)	0.08
CRP (mg%)	121.9 (145.2)	134 (55.4)	61 (184.6)	0.41
**Scores**				
PAS score (/10)	5 (2.5)	5 (2)	4.5 (4)	0.051
Alvarado (/10)	5 (3.5)	5 (3)	4.5 (4.2)	0.37
Duration of hospital stay (d)	6 (4)	7 (4)	3.5 (3.75)	0.018

Data are shown by the median value and (interquartile range)

There was no mortality.

## Discussion

Our study confirms that acute appendicitis in preschool children is rare, accounting for less than 10% of all pediatric cases [3]. In our series, diagnosis of appendicitis was made after a median period of 48 h following the apparition of the first symptoms, the majority of the patients having been assessed ambulatory and discharged with a diagnosis of a banal viral infection, in particular gastro-enteritis or urinary tract infection. This is in line with previous reports indicating that diarrhea is a frequent symptom of acute appendicitis explained by the effect of abdominal infection on intestinal motility [7, 16, 17].

Indeed, diagnosis of appendicitis in preschool children is challenging and burdened by a high rate of misdiagnosis resulting from atypical clinical signs and by trivialization of abdominal pain in this age group [3]. On the contrary to school children and adolescents, younger children do not present the classical clinical picture with initial anorexia and peri-umbilical pain that migrates in the right fossa iliaca, vomiting and fever [1]. According to that, in our series, the only constant symptom on admission was abdominal pain that was diffuse in the majority of cases. Fever, anorexia and transit alteration were not observed in all patients.

Previous studies have shown that complications due to appendicitis are more frequent and more severe in children than in adults. Furthermore, patients with complicated appendicitis are more likely to be under 5 years of age and to have had symptoms for a period exceeding 24 h, compared to patients with uncomplicated appendicitis [18]. Our results are consistent with that. The non-specific clinical presentation of acute appendicitis in young children is thought to be responsible for diagnosis delay and therefore for higher rate of complications in this age group, as it has been shown in patients younger than 5 years of age [3]. This fact is illustrated in our cohort in whom a high rate of peritonitis (72%), appendicular abscess (41%), and appendicular perforation (38%) was observed.

This is in line with previous reports showing that delayed diagnosis expressed by the duration of pre-admission symptom is associated with appendix perforation. Indeed, after 36 h of symptoms, the risk of perforation increases by 5% each 12 h [19].

Diagnosis difficulties in children with appendicitis have led to the attempt to use of scoring systems. In this study, we applied retrospectively the Alvarado- and the PAS scores but found the results not contributive, in accordance with previous report [20]. In a large previous prospective study, both scoring systems were assessed and compared but both scores gave a specificity lower than 60% and none had a sufficient predictive value for the diagnosis of acute appendicitis [6]. Since most items entered for score calculation are clinical signs that have a low incidence in pre-school children, patient age is clearly expected to influence the predictive value of Alvarado score and PAS. Hence, the controversial results reported on appendicitis scores performance in children may be explained by the important age variability in the different large series reported [6, 13, 15].Besides the lack of specificity of clinical signs for diagnosing acute appendicitis in young children, laboratory examinations and imaging are also imperfect diagnosis tools yet. Nevertheless, the literature admits that the elevation of biological markers such as WBC count, PN count and CRP is often observed in acute appendicitis, but it lacks of specificity, especially when it is isolated [21, 22].

In our patients, first abdominal ultrasound was suggestive of appendicitis in only 65.5% of the cases. In most of children in whom abdominal ultrasound was negative, appendix was either not- or incompletely visualized. A frequent cause for that is ectopic position of the appendix. This explains the relative high rate of abdominal CT-scanner that had to be performed in this cohort. Nevertheless, abdominal ultrasound should, due to the possibility to easily repeat examinations, remain the first choice and the most frequently performed examination for the diagnosis of appendicitis in the pediatric population [23, 24]. The role of magnetic resonance imaging for the diagnosis of acute appendicitis in young children remains to be established [25].

An objective of this study was to analyze the influence of age on outcome variables among the group of preschool children. Owing to the age distribution in our series, we considered 2 patients groups younger or older than 48 months of age. Our results show that, as expected, the youngest had the longest duration of clinical symptoms before diagnosis and treatment. The youngest had also more frequent alteration of the general state, indicating systemic involvement of the disease and less frequent pain in the right fossa iliaca. This latter might be explained by a preponderance of visceral abdominal pain in contrast to parietal abdominal pain in younger children [26] and not by appendix localization, in particular in retro-caecum position that was equally present in both groups.

Children younger than 48 months of age tended to have higher central temperature at admission, as a sign of higher systemic repercussions of the abdominal infection in this group. They also showed significantly higher heart rate that besides the fact that this decreases physiologically with age may be explained by the combination of higher central temperature and hemodynamic adaptation to severe infection.

Interestingly, our results show that in younger children, there was dissociation in the inflammatory response with significantly lower WBC- and PN counts in combination with higher CRP levels than in older children. Indeed, PN count at admission correlated positively with age and CRP negatively, and WBC- and PN counts were significantly lower in children younger than 48 months than in the older ones.

This suggests age-related WBC and PN migration with impaired recruitment from the bone marrow into the circulation in younger children in spite of an important inflammatory response to the bacterial infection reflected by the induction of high levels of CRP in the liver [27]. This observation points out the absolute necessity to measure blood levels of CRP together with WBC count in order not to misinterpret normal or low WBC counts that may consolidate the presumption of banal viral infection, and especially as a large literature review concluded that lower WBC count decreased the likelihood of appendicitis in children [28]. Low WBC- and PN counts in young children had certainly also contributed to the underscoring and lack of specificity of both the Alvarado score and PAS in our patient population.

In our series, and in accordance with previous reports [2, 3, 12], younger children developed a higher rate of complications such as perforation with purulent peritoneal liquid that is, as discussed above, the result of prolonged disease course. However, the rate of other complications such as abscess, ileus or sepsis was not different between groups.

### Limitation section

This study has several limitations related to its retrospective design and to the small patient cohort analyzed according to the rarity of the disease in the elected age group.

## Conclusions

This study confirms that acute appendicitis in children less than 6 years of age is a rare condition and is still related to a high risk of morbidity, especially appendix perforation, due to the diagnostic delay. This latter in turn is the consequence of non-specific symptoms and the non-pathognomonic clinical-and complementary examination results and increases with younger age.

Less specific, trivialized abdominal pain and dissociation of the inflammatory markers with lower leucocyte- and neutrophil counts and higher C-reactive protein levels in young children contribute to the diagnosis trap of acute appendicitis in preschool children.The greatest caution is therefore mandatory when evaluating a young child with acute abdominal pain and the question of whether it could be acute appendicitis systematically addressed.

## Data Availability

The datasets used and/or analyzed during the current study are available from the corresponding author on reasonable request.

## Abbreviations

bpm: beat per minute
CRP: C-reactive protein
CT: Computed tomography
IQR: Interquartile range
PN: Polynuclear neutrophils
PAS: Pediatric appendicitis score
WBC: White blood cells
